# Degradation Study on Sulfasalazine and a Validated HPLC-UV Method for its Stability Testing

**DOI:** 10.3797/scipharm.1311-15

**Published:** 2014-01-12

**Authors:** Balraj Saini, Gulshan Bansal

**Affiliations:** Department of Pharmaceutical Sciences and Drug Research, Punjabi University, Patiala – 147002, India.

**Keywords:** Sulfasalazine, Forced degradation, HPLC, ICH, Stability-indicating

## Abstract

Sulfasalazine (SSZ) was subjected to degradation under the conditions of hydrolysis (acid, alkali, and water), oxidation (30% H_2_O_2_), dry heat, and photolysis (UV-VIS light) in accordance with the ICH guidelines. An RP-HPLC method was developed to study the degradation behavior. No degradation was noted under any condition except alkaline hydrolysis where SSZ was degraded to a single minor product. SSZ was optimally resolved from this product on an XTerra^®^ RP18 column with a mobile phase composed of methanol and an ammonium acetate buffer (10 mM, pH 7.0) (48:52, v/v) delivered at a rate of 0.8 mL/min in an isocratic mode. The method was validated and found to be linear (r^2^=0.99945), precise (%RSD <2), robust, and accurate (94–102%) in the concentration range of 0.5–50 μg/mL of SSZ. The PDA analysis of the degraded sample revealed the SSZ peak purity to be 998.99 and the drug peak eluted with a resolution factor of >2 from the nearest resolving peak, indicating the method to be selectively stability-indicating for the drug analysis. The method was applied successfully for the stability testing of the commercially available SSZ tablets that were under varied ICH-prescribed conditions. An explanation for the unusual stability of the drug when exposed to acidic hydrolysis, despite the presence of the sulfonamide linkage, is also discussed.

## Introduction

**Fig. 1:**
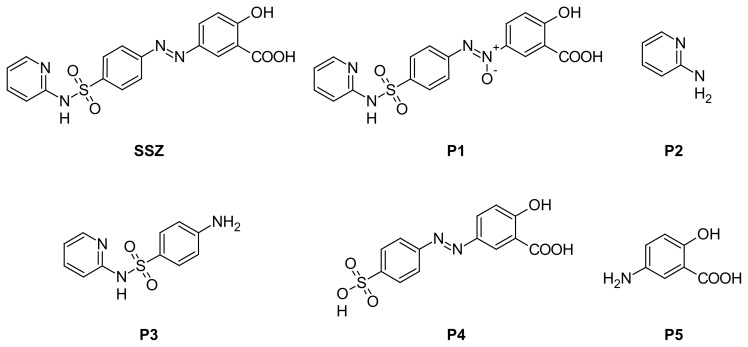
Sulfasalazine (SSZ) and its probable degradation products (P1–P5).

Sulfasalazine (SSZ), 2-Hydroxy-5-[(*E*)-2-{4-[(pyridin-2-yl)sulfamoyl]phenyl}diazen-1-yl]-benzoic acid (Fig. 1), is a disease-modifying, anti-rheumatic drug which helps to reduce joint pain, swelling, and stiffness [1]. Its recommended dose starts at 500 mg once daily and is increased up to 2–3 g per day divided into 2–3 doses over 3–11 months to achieve therapeutic efficacy [2, 3]. The presence of an azo and a sulfonamide linkage in the chemical structure of the drug makes it prone to degradation during the various stages of formulation development, leading to the probable appearance of some degradation-related impurities in the final product. While the sulfonamide linkage is susceptible to hydrolysis in acidic medium [4] to form the corresponding sulfonic acid derivative and amine, the azo group can undergo chemical changes under hydrolytic, photolytic, and oxidative conditions to form different products [5–8]. Based on these chemical susceptibilities, the suspected degradation products of SSZ are given in Fig. 1. However, it is reported that sulfonation in an azo group-containing molecule stabilizes the azo linkage [5]. Though some process-related impurities of SSZ are official in the British Pharmacopoeia [9], there is no report available in the literature on the degradation products of the drug. The International Conference on Harmonization (ICH), through its guidelines Q1A (R2) and Q1B [10, 11], requires the conduct of forced degradation studies to characterize all of the possible degradation products under varied conditions such as light, heat, humidity, acid/base hydrolysis, and oxidation in order to develop a stability-indicating assay method (SIAM) for the stability testing of a drug substance or product. An extensive literature search has revealed that several analytical methods were reported in the 1990s for the analysis of SSZ alone [12, 13], in the formulations [14], and in the presence of metabolites [15–21]. Peel et al., 1994 [22] and Coward et al., 1995 [23] had reported interference of SSZ with the HPLC assay of urine and 5-hydroxyindole-3-acetic acid, respectively. The most recent report on any analytical method for SSZ was published in 2011 where an HPLC method was developed for the quantification of SSZ along with mebeverine, mesalazine, and dispersible aspirin [24]. A thin-layer chromatography method was also reported for the characterization of process-related impurities in SSZ [25]. However, none of the reported study was carried out in accordance with the ICH guidelines that help in establishing the stability-indicating nature of the analytical method. Moreover, there is no single analytical method available for the quantification of SSZ in the presence of its degradation products. Hence, the present study was undertaken to carry out the ICH-prescribed forced degradation study on SSZ to characterize the degradation products and to develop and validate a stability-indicating HPLC method. Finally, the method was applied to stability testing of the commercially available SSZ tablets.

## Experimental

### Chemicals and Reagents

SSZ was procured as a gift sample from Cadila Pharmaceuticals Ltd. (Ahmadabad, India). Methanol (HPLC grade), sodium hydroxide (NaOH) pellets, dimethyl sulfoxide (DMSO), and acetic acid (all AR grade) were purchased commercially from Merck (Mumbai, India). The solutions were prepared using HPLC grade water prepared by the Lab Pure Water purification System (Bio-Age, SAS Nagar, India) in the laboratory. The SSZ tablets (SAZO^™^ 500, Wallace Pharmaceuticals Pvt. Ltd, Goa, 500 mg per tablet) were purchased from the local pharmacy store.

### Instruments

A high precision water bath and a hot air oven (Narang Scientific Works, India) with temperature control of ± 1 and ± 2°C, respectively, were used for the hydrolytic and thermal degradation studies. Photodegradation was carried out in a photostability chamber (Binder, Germany) equipped with a light bank consisting of two UV (OSRAM L73) and four fluorescent (OSRAM L20) lamps and capable of controlling the temperature and humidity in the range of ± 2°C and ± 5% RH, respectively. The light system complied with option 2 of the ICH guideline, Q1B [11]. At any given time, the UV energy and visible illumination were tested with a calibrated radiometer (206, PRC Krochmann GmbH, Germany) and a calibrated lux meter (ELM 201, Escorp, India). The HPLC system consisted of a 2487 UV/Visible dual wavelength detector, a Rheodyne manual injector, and two 515 pumps (Waters, Milford, USA). A reversed-phase C18 column (250 mm × 4.6 mm i.d., 5 μm) (XTerra^®^, Waters, Milford, USA) was used for the chromatographic separations. Data were acquired and processed with Empower 2 software. PDA analysis was carried out on an Agilent HPLC system (Agilent 1200 Series, Santa Clara CA, USA).

### Preparation of Solutions and Reagents

The NaOH and HCl (0.1, 1, 2, and 5 N) solutions were prepared to their approximate strengths in water. A 40% (v/v) solution of DMSO was prepared in water and used as diluent. A stock solution of SSZ (1 mg/mL) prepared in the diluent was serially diluted with the diluent to obtain standard solutions in the concentration range of 0.5–50 μg/mL.

### Forced Degradation Study

The hydrolytic degradation study was carried out in water, in 0.1, 1, 2, and 5 N HCl, as well as NaOH at 85°C for 24 h. Oxidative degradation was carried out in 30% H_2_O_2_ solution at room temperature for 24 h. For thermal degradation, the drug was sealed in amber-colored borosilicate glass vials and placed in the hot air oven maintained at 50°C for 31 days. The photolytic studies were carried out by exposing a thin layer of the solid drug in a Petri dish, as well as solutions of the drug in 1 N HCl, water, and 1 N NaOH to the light in the photostability chamber for 10 days, during which the total fluorescent light exposure was equaled to 1.2 million lux h and UV exposure was 200 watt hm^−2^. A parallel set was kept in the dark under the similar conditions for the same duration to serve as a dark control. Each degraded drug solution was diluted up to 10 times with the diluent for HPLC analysis. The solid degraded drug samples were rendered into solution using the diluent to achieve a concentration of 0.01% (w/v). The alkali and acid samples were neutralized with acid or alkali, before dilution.

### Separation Method

The chromatographic separation of SSZ from its degradation product was achieved on the XTerra® RP18 column (250 mm × 4.6 mm i.d., 5μm) using a mobile phase composed of methanol and ammonium acetate (10 mM, pH 7.0 with acetic acid) (48:52, v/v) at a flow rate 0.8 mL/min. The eluent was detected at 360 nm and injection volume was fixed at 20 μL.

### Method Validation

The method was validated by evaluating various validation parameters such as linearity, precision, accuracy, robustness, LOQ, and LOD in accordance with the ICH guidelines, Q2(R1) [26]. The linearity was evaluated by analyzing standard solutions of SSZ (0.5–50 μg/mL) in the order of increasing concentrations. The standard solutions were prepared in triplicate to generate one calibration curve from each set of standard solutions and subjected to linear regression analysis to calculate slope, intercept, and determination coefficient (r^2^). The intraday and interday precision were determined by analyzing each of the three concentrations (1, 5, and 25 μg/mL) of SSZ, respectively, on the same day and on three different days. Each concentration was analyzed six times consecutively and precision was expressed as %RSD of each calculated concentration. For the evaluation of accuracy, the drug concentration in the degraded drug solution was fortified by separately mixing equal volumes of the standard drug solutions in the concentrations 1, 5, and 25 μg/mL so that the drug concentration was fortified by 0.5, 2.5, and 12.5 μg/mL, respectively. The same degraded solution was also mixed with equal volumes of diluent to serve as an unfortified solution. The drug concentration in each fortified and unfortified solution was determined (n=3) and accuracy was expressed as the percent recovery of the fortified drug concentration vis-à-vis the unfortified. The LOD and LOQ were determined by the calibration curve method using the following equations [26]:

LOD=3.3(σ/S);         LOQ=10(σ/S)

where σ = the standard deviation of the response; S = the slope of the calibration curve. The slope S and *σ* were estimated from the calibration curve of the analyte. Subsequently, the drug solution of the calculated LOD and LOQ was prepared and analysed (n=10). The %RSD of the peak areas of both the LOD and LOQ was calculated. The robustness of the developed HPLC method was determined by making small, but deliberate changes in various chromatographic parameters of the optimized procedure like the composition of the mobile phase, λ_max_, flow rate, and column brand. The standard solution of SSZ (20 μg/mL) was analysed (n=3) at each varied chromatographic condition. The recovery of the SSZ was calculated and a change in RT was noted vis-à-vis the optimized chromatographic conditions. The selectivity of the method was established by PDA analysis of the degraded drug solution using the same chromatographic conditions as for the HPLC-UV analysis.

### Stability Testing of SSZ Tablets

A blister strip of SSZ tablets was exposed to accelerated conditions of 40°C/ 75% RH in the photostability chamber for 6 months. Another strip was kept in the dark under similar conditions for 6 months. For thermal degradation, the tablets were placed in a hot air oven maintained at 50°C for 31 days. The real time stability samples were generated by keeping the tablets at room temperature (30 ± 5°C and 65% RH) for 20 months. A control sample was kept in a refrigerator at 4°C. Each stability sample was analyzed by the validated HPLC method to quantify SSZ. The packed tablets from each stability condition were processed as follows. Ten tablets were weighed, powdered, and a quantity of the powder equivalent to 10 mg of SSZ was transferred into a 10-mL measuring flask. The powder was mixed with 8 mL of diluent and sonicated for 10 min. The contents were brought to room temperature and the volume was adjusted up to the mark. The resultant stock solution was diluted with the diluent to a drug concentration of 50 μg/mL. The sample solutions were filtered through a 0.45 μm membrane and analyzed (n=3) for the content of SSZ using the validated HPLC method. The content of SSZ was reported as the label claim.

## Results and Discussion

### Method Development

The UV absorption spectra of SSZ in the diluent revealed 360 nm as the absorption maxima and hence, this was selected as the detection wavelength on the HPLC detector. All chromatographic methods reported in the literature were found to employ a mobile phase composed of a phosphate buffer which is not compatible with the LC-MS system. However, the present study is targeted at the characterization of the degradation products through isolation followed by spectral analysis or through LC-MS studies of the degraded samples. Hence, to make the chromatographic conditions compatible with the LC-MS system in order to characterize the non-separable degradation products through LC-MS data, the phosphate buffer was replaced with an ammonium acetate buffer. The method development was initiated using a mobile phase composed of methanol: water (60:40, v/v) flowing at a rate of 1 mL/min on a C18 column (150 × 4.6 mm, 5 μm). The drug was eluted as an unsymmetrical peak (As= 0.33) along with a large hump at 30–35 min. An increase in the proportion of methanol up to 85 % did not improve the symmetry of the drug peak (As= 0.38), whereas increasing the flow rate to 1.5 mL min^−1^ slightly sharpened the peak (As= 0.558), but the column back pressure was also excessively increased. Taking into account the pKa values of SSZ (2.4, 9.7, and 11.8), the pH of the mobile phase (70% methanol in water) was adjusted at 3.0 with acetic acid. It resulted in a symmetrical drug peak (As= 1.111), but the retention time (RT) was not reproducible probably due to the volatility of acetic acid. Replacement of the aqueous component of the mobile phase with an ammonium acetate buffer (pH 3.0) and decreasing the flow rate to 0.5 mL/min eluted the drug with a tailing factor of 1.33. A gradual decrease in methanol content to 55 % with a concomitant increase in the buffer content and increasing the column length to a 250 mm column resulted in a symmetrical drug peak (As= 0.925) at 24.5 min. However, these chromatographic conditions were not able to resolve the drug and the degradation peak in the alkali-degraded sample. Though the resolution was improved by using 45% methanol, it also resulted in the broadening of the drug peak. A stepwise increase in the pH of the mobile phase to 7.0 with NH_4_OH eluted the drug peak at 16.5 min and the degradation product at 12.7 min. A small increase in methanol content to 48 advanced the two peaks to 13.7 and 9.9 min, respectively, and also improved the peak shape (As= 0.961 and 0.714, respectively). Hence, these chromatographic conditions were used for the analysis of the forced degradation samples and validated according to the ICH guidelines.

### Degradation Behavior

**Fig. 2:**
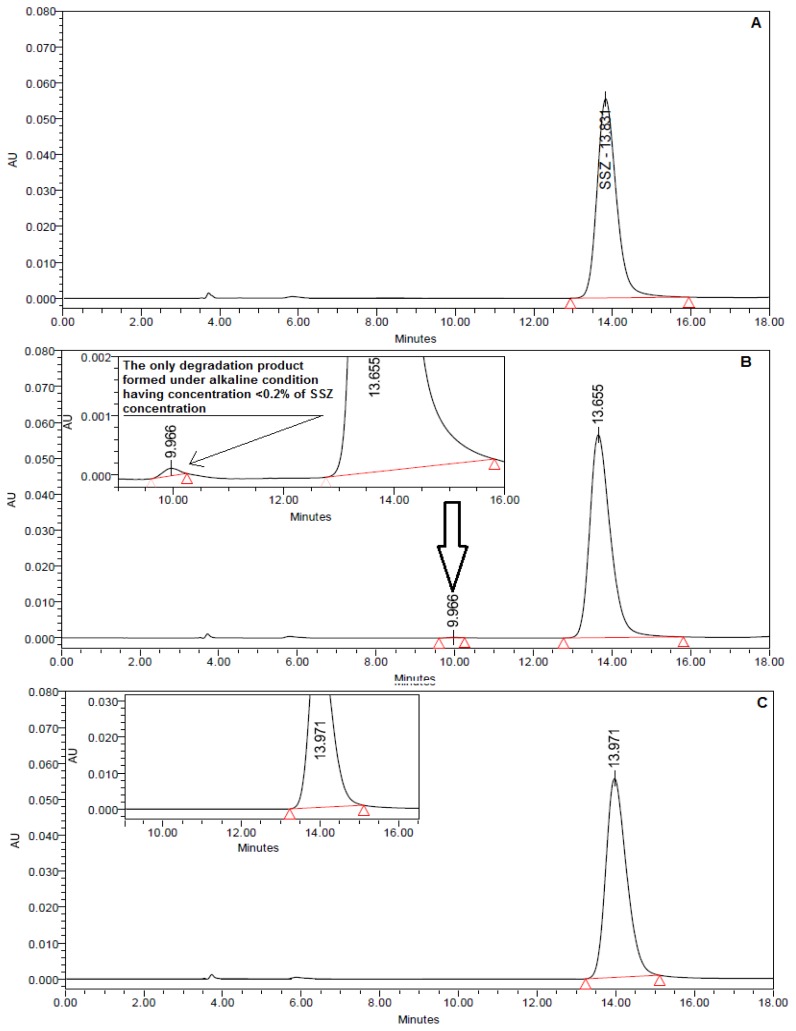
HPLC-UV chromatograms. (A) SSZ standard solution (20 μg/ml), (B) alkali-degraded sample (5N) showing little degradation, and (C) representative stability testing sample.

**Fig. 3:**
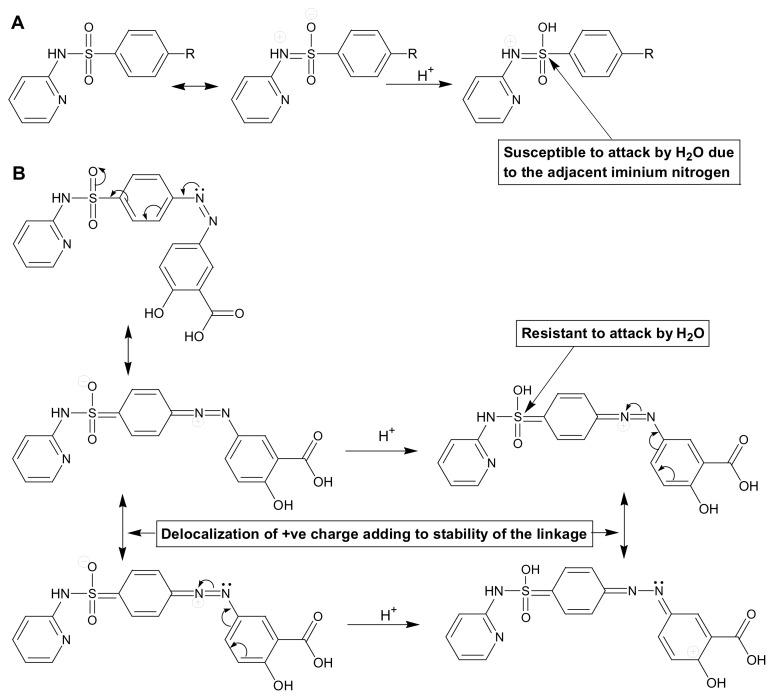
Relative susceptibility of sulphonamide linkage in normal aryl sulfonamides (A) and in sulfasalazine (B).

No degradation was noted in 0.1 N HCl, 0.1 N NaOH, and water at 85°C for 24 h. The drug also remained stable in 2 N HCl and 2 N NaOH at 85°C for 24 h, as no degradant peak was noted in the HPLC chromatograms. A further increase in HCl strength to 5 N at 85°C for 24 h also did not cause any change in the chromatogram. However, a degradant was eluted as a minute peak (area <0.2% of the SSZ peak) just before the drug peak in the drug solution in 5 N NaOH subjected to 85°C for 24 h, which could be noted only after zooming in to the chromatogram (Figs. 2A and B). The same peak was not noted in either the 5 N NaOH blank or in the standard drug solution, which suggested it to be a degradation product. However, the degradation product formed under the alkaline conditions was not characterized due to the very low concentration (<0.1%). The drug was found to be stable under all other conditions such as dry heat, photolysis, and oxidation. It suggested that the drug is stable in varied chemical environments and is not liable to undergo any chemical changes during formulation and/or shelf life. This unusual stability of the drug despite the presence of the N-aryl sulfonamide linkage, which is otherwise susceptible to acidic hydrolysis, is attributed to the presence of a diazo group at *para*-position with respect to the sulfonyl group. The delocalization of electrons initiated by the non-bonding electrons of the nitrogen make the sulfonyl oxygen highly nucleophilic, which is protonated by the acidic medium and no electrophilic center is generated adjacent to the sulfonyl sulfur making the latter resistant to attack by the oxygen of the water. Further, the *+ve* charge on the nitrogen is resonance-stabilized by delocalization into the adjacent phenyl ring. The course of the reaction leading to the attack of water for hydrolysis of the sulfonamide linkage in acidic medium in normal aryl sulfonamides as well as SSZ is outlined in Fig. 3.

### Method Validation

The relation between the peak area and the concentration of SSZ was found to be linear in the concentration range of 0.5–50 μg/mL. The mean (± SD) slope, intercept, and r^2^ were 133635 (± 7697.5), −136098.5 (± 13324.6), and 0.9995 (± 0.00007), respectively. The LOD and LOQ were found to be 0.3 and 1.0 μg/mL, respectively, with RSD values of 1.98% and 1.01%. Good recoveries (94.18–102.30%) of SSZ at each fortification level were achieved with the RSD less than 1.0% (Table 1). It suggested that the method is sufficiently accurate for the quantification of SSZ. The method was found to be sufficiently precise with the RSD for the interday and intraday precision found to be less than 1.7% (Table 1). No significant variation in the calculated drug concentration was observed on any day. It showed that the method was sufficiently precise for determining the drug concentrations. The method was found to be robust as the % change in the calculated SSZ content and change in RT of SSZ were not significant after deliberate changes were made to the method variables including λ_max,_ composition of the mobile phase, pH of mobile phase, flow rate, and column (Table 2). The PDA analysis of the degraded drug solution revealed the drug peak purity to be 998.99, which indicated it to be selective for the drug. Further, the drug peak resolved from the nearest peak with a resolution factor of >2 which established the method to be selectively stability-indicating.

### Stability Testing of SSZ Tablets

The method was applied successfully to the stability testing of the SSZ tablets. No significant changes in the content of SSZ in the tablets exposed to varied conditions was noted (Table 3). The drug peak in all stability samples was found to be pure (Table 3) and no extra peak was noted in any chromatogram (Fig. 2C). Hence, the tablets were found to be stable under all stability conditions.

## Conclusion

SSZ was subjected to the various forced degradation conditions like hydrolysis, oxidation, dry heat, and photolysis in accordance with the ICH guidelines and found to be stable under all conditions except extreme alkaline conditions. An RP-HPLC method was developed using an XTerra^®^ RP18 column (4.6 mm × 250 mm; 5 μm) with a mobile phase composed of methanol and an ammonium acetate buffer (10 mM, pH 7.0) (48:52, v/v) delivered at a rate of 0.8 mLmin^−1^ in an isocratic mode and detection was carried out at 360 nm. The method was found to be linear (r^2^=0.99945), precise (%RSD < 2), robust, and accurate (recovery 94–102%) in the concentration range of 0.5–50 μg/mL of SSZ under various validation parameters. The method was selective and stability-indicating for the drug analysis, as indicated by the PDA analysis (purity factor 998.99) of the degraded sample. The method was applied successfully for the stability testing of the commercially available SSZ tablets under the varied ICH-prescribed conditions. The drug was found stable in all stability conditions.

## Figures and Tables

**Tab. 1:** Accuracy and intraday and interday precision of the method

Accuracy studies		

Conc. Added (μg/mL)	Conc. Found (μg/mL)	% Recovery (Mean±SD; %RSD)
1.0	1.01	101.7 ± 0.82; 0.81
5.0	4.91	94.16 ± 0.92; 0.98
25.0	25.07	100.30 ± 0.1; 0.14

**Precision studies**		

Actual conc. (μg/mL)	Calculated conc. (μg/mL) ± S.D.;%RSD	

	Intra-day (n = 6)	Inter-day (n = 3)

1.0	1.10 ± 0.01; 0.91	1.13 ± 0.01; 0.88
5.0	5.13 ± 0.09; 1.75	5.16 ± 0.08; 1.55
20.0	19.76 ± 0.14; 0.71	19.72 ± 0.04; 0.25

**Tab. 2:** Robustness study

Robustness Parameter	RT	ΔRT	Content of SSZ (μg/mL)	% Difference in content
Optimized condition	13.40	–	18.82	–
Mobile phase B:M (50:50)	12.39	−1.01	18.65	0.87
Mobile phase B:M (46:54)	15.64	2.24	18.49	1.72
Mobile phase (pH 6.9)	15.89	2.49	18.17	3.42
Mobile phase (pH 7.1)	16.04	2.64	18.15	3.55
Flow Rate (0.7 ml min^−1^)	15.49	2.09	18.11	3.79
Flow Rate (0.9 ml min^−1^)	12.15	−1.25	18.12	3.69
λ_max_ (355 nm)	13.83	0.43	18.16	3.51
λ_max_ (365 nm)	13.74	0.34	18.19	3.37
Column Kromasil	13.65	0.25	18.71	0.58
Column Inertsil	13.97	0.57	19.70	−4.69

B…Ammonium acetate Buffer; M…Methanol

**Tab. 3:** Stability testing data of SSZ tablets

Stability condition	Content of SSZ (mg per tablet)	Recovery (%)	Peak Purity
Control (4°C)	498.5	100.0	998.59
Thermal (50°C; 31 days)	495.6	99.4	999.05
Photostability (40°C/75% RH, UV-VIS; 6 months)	497.4	99.7	999.21
Accelerated (40°C/75% RH; 4 months)	494.9	99.3	998.67
Real time (30 ± 5°C, 65 ± 5% RH; 20 months)	488.9	98.1	998.45
